# Limb articulation of biological motion can induce illusory motion perception during self-motion

**DOI:** 10.1177/20416695241246755

**Published:** 2024-05-27

**Authors:** Anna-Gesina Hülemeier, Markus Lappe

**Affiliations:** Institute for Psychology, 9185 University of Münster, Münster, North-Rhine Westphalia, Germany; Institute for Psychology, 9185 University of Münster, Münster, North-Rhine Westphalia, Germany

**Keywords:** speed estimation, point-light walkers, limb articulation, scrambled, biological

## Abstract

When one walks toward a crowd of pedestrians, dealing with their biological motion while controlling one's own self-motion is a difficult perceptual task. Limb articulation of a walker is naturally coupled to the walker's translation through the scene and allows the separation of optic flow generated by self-motion from the biological motion of other pedestrians. Recent research has shown that if limb articulation and translation mismatch, such as for walking in place, self-motion perception becomes biased. This bias may reflect an illusory motion attributed to the pedestrian crowd from the articulation of their limbs. To investigate this hypothesis, we presented observers with a simulation of forward self-motion toward a laterally moving crowd of point-light walkers and asked them to report the perceived lateral speed of the crowd. To investigate the dependence of the crowd speed percept on biological motion, we also included conditions in which the points of the walker were spatially scrambled to destroy body form and limb articulation. We observed illusory crowd speed percepts that were related to the articulation rate of the biological motion. Scrambled walkers also produced illusory motion but it was not related to articulation rate. We conclude that limb articulation induces percepts of crowd motion that can be used for interpreting self-motion toward crowds.

Optic flow is the expanding pattern of visual motion of the surrounding scene that one experiences during self-motion (Gibson, 1950). It plays a pivotal role in self-motion perception. The moving points within the optic flow field result from the translational and rotational components of the motion of the eye in the three-dimensional space during self-motion. Mathematical decomposition of the optic flow provides access to self-motion perception, like the estimation of the direction of self-motion or heading (Longuet-Higgins & Prazdny, 1980). Computational models and empirical observations prove the feasibility of this analysis in rigid environments (e.g., Cutting, 1986; Cutting et al., 1992; Li et al., 2006; Royden et al., 1992; Warren & Hannon, 1988; Warren et al., 2001). But any independent motion of either objects (Layton & Fajen, 2016; Li et al., 2018; Royden & Hildreth, 1996; Warren & Saunders, 1995) or human beings (Hülemeier & Lappe, 2020; Koerfer & Lappe, 2020; Mayer et al., 2021; Riddell & Lappe, 2017, 2018; Riddell et al., 2019) in the scene violates the rigidity assumption and, due to this violation, creates biases in self-motion perception, or heading estimation.

The term biological motion refers to the distinctive pattern of articulated motion of the arms and legs during the gait cycle of a walking human that one experiences when seeing another person walking. It is typically studied by presenting point-light walkers in which only points on the joints are shown (Johansson, 1973), thereby reducing the visual information to the articulation of the limbs (see Figure 1, left). Such a point-light walker consists of 12 points representing the major joints like shoulders, elbows, hands, hips, knees, and feet. As a point-light walker moves across the scene, the motion pattern of the points encompasses both body translation and limb articulation, which together convey crucial information about the walker's speed and direction (Giese & Lappe, 2002; Masselink & Lappe, 2015; Thurman & Lu, 2016).

**Figure 1.**
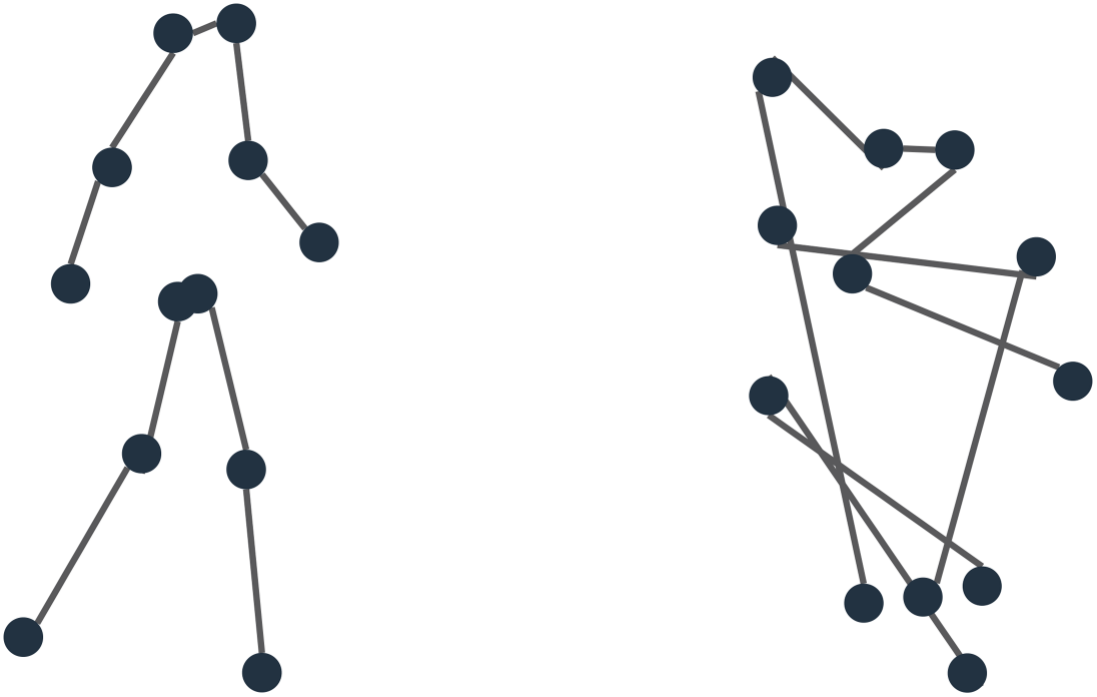
Example of a normal (left) and scrambled (right) point-light walker. The dots are connected to assist in recognizing the limb structure and body form of the point-light walker. In the scrambled point-light walker, the limb structure and body form are destroyed.

Optic flow and biological motion are experienced together when one moves through a scene that contains other pedestrians. Theoretical considerations on the pattern of motion received in this could regard the interplay between biological motion from the pedestrian and the optic flow field from self-motion as two-sided. On the one hand, biological motion disrupts the rigidity assumption needed for optic flow analysis by introducing independent motion (i.e., the translation of the other walkers) into the flow field, resulting in errors in heading estimation (Hülemeier & Lappe, 2020; Koerfer & Lappe, 2020; Riddell & Lappe, 2017, 2018; Riddell et al., 2019). On the other hand, biological motion contains valid cues about the walkers’ motion (Giese & Lappe, 2002; Masselink & Lappe, 2015), counteracting the heading errors and enhancing the accuracy of self-motion perception (Hülemeier & Lappe, 2020; Riddell & Lappe, 2018).

As empirical studies confirmed, the combination of limb articulation and body translation as in natural locomotion allows proper heading perception (Hülemeier & Lappe, 2020; Riddell & Lappe, 2018) despite the massive perturbations in the flow field caused by these motion components. Interestingly, when the walkers only move their limbs but do not translate through the scene, as if on a treadmill, significant heading errors occur (Hülemeier & Lappe, 2020; Riddell & Lappe, 2018). Proper heading estimates in natural locomotion, therefore, suggest that limb articulation transmits some information to heading estimation that normally compensates for the translation of the walkers but, in the absence of this translation, introduces errors.

The latter observation contradicts considerations based on a pure optic flow perspective. From a pure optic flow perspective, limb articulation coupled with crowd speed violates the rigidity assumption much stronger compared to articulating walkers without crowd speed. The rhythmic gait pattern of the articulating limbs creates a kind of balanced noise so that self-motion perception should be less accurate but not strongly biased. Thought further, participants should therefore be able to accurately determine their self-motion, thus accurately discerning the absence of crowd speed. Considering the observed self-motion biases in the presence of only-articulating walkers (Hülemeier & Lappe 2020, 2023; Koerfer & Lappe, 2020; Riddell & Lappe, 2017, 2018), these heading errors may be related to backscroll illusion (Fujimoto, 2003; Fujimoto & Sato, 2006; Fujimoto & Yagi, 2008): In the backscroll illusion, when a walker articulates as if on a treadmill in front of a background, this background is perceived as moving in opposite direction than the walker faces. During self-motion, this illusory motion might indicate an additional component of self-translation or self-rotation of the observer. When the walkers are the only visible elements in the scene, such an illusory self-motion percept might affect the heading perception from the optic flow (Hülemeier & Lappe, 2020, 2023). This consideration, in turn, implies that the participants may alternatively deduce crowd speed from the limb articulation pattern due to their knowledge of the natural connection between limb articulation and body translation (Masselink & Lappe, 2015). Crowd speed estimates would then indicate an illusory crowd motion perception. Our experiment was intended to test this hypothesis.

A subsequent question regarding the influence of biological motion on optic flow processing is whether the influence arises from the limb articulation coupled to the body form of a walker or from the motion trajectories of the individual points. Our experimental design, therefore, seeks opportunities to disentangle the influence of limb articulation and the motion trajectory of individual points. Using point-light walkers allows for the decoupling of limb articulation from the motion of the individual points. Scrambling the point-light walkers (Figure 1, right) relocates the position of the points without changing the point's motion trajectory. In this way, scrambling destroys the form of the human body and the relationship between limbs but retains motion cues of the individual points (Cutting, 1981). Employing scrambled walkers can test whether observed responses are specific to biological motion or not.

Previous studies employed normal walkers who either moved naturally or only articulated their limbs as if on a treadmill with scrambled variants of those stimuli (Hülemeier & Lappe, 2020; Riddell & Lappe, 2018). In the natural locomotion condition, heading errors were larger for scrambled than for biological displays. Conversely, in the condition where the walkers only articulated their limbs, heading biases were smaller for scrambled compared to normal displays. These results hint that limb articulation either coupled with matching crowd speed or presented in isolation (natural locomotion vs. only-articulation) may cause different degrees of backscroll illusions and differing influences on heading estimation during self-motion.

In light of these biases produced by limb articulation, we investigate the extent to which limb articulation of a walker, coupled with self-motion simulation, produces an illusory percept of crowd motion. When point-light walkers articulate their limbs without translating, we hypothesize that observers will falsely attribute part of their self-motion to a perceived crowd speed that physically does not exist. Due to the heading biases in the presence of biological and scrambled walkers (Hülemeier & Lappe, 2020; Riddell & Lappe, 2018), we expect this effect to be more pronounced for biological walkers compared to scrambled ones.

## Methods

### Sample

Thirty-four volunteers from the University of Münster participated in the experiment (female: 25, male: 9). Age ranged between 18 and 30 (*M *= 22.18, *SD *= 3.26). Their visual acuity was normal or corrected-to-normal. Participation was voluntary, anonymous, and compensated by either course credits or 10€/h. The study was approved by the ethics committee of the Department of Psychology and Sport Science at the University of Münster.

### Experimental Settings

The experiment took place in a darkened laboratory room. Stimuli were generated in MATLAB (version R2023a, The MathWorks, Natick, MA) with the OpenGL libraries add-ons of the Psychophysics toolbox (version 3.0.19, Kleiner et al., 2007). We connected a Lenovo IdeaPad laptop to a VDC Display Systems Marquee 8500 projector to project the stimuli onto a 250 cm × 200 cm backlit screen. The screen resolution was 1024 × 768 pixels at a frame rate of 60 Hz. During the stimulus presentation, subjects sat about 100 cm away from the screen. The display had a size of 102° × 90°.

### Experimental Scene

We simulated self-motion at a natural constant speed (1.1 m/s) on a linear path towardt a crowd of eight life-sized point-light walkers. Heading of the simulated self-motion varied within 12° to the left and right of the screen center across trials. Walkers were constructed from motion-tracking data of a naturally walking person (de Lussanet et al., 2008). Each walker consisted of 12 white points whose positions corresponded to the main joints of the human body (shoulders, elbows, hips, wrists, knees, and ankles). The walkers faced collectively either to the left (−90°) or right (90°). Point-light walkers were placed on an invisible ground plane. In the virtual scene, half of the group was placed 7 m and 9 m from the observer, and the other ones 14 to 18 m. Objects that are placed at different distances from the observer give rise to motion-parallax (Hanes et al., 2008; Koenderink, 1986). Motion-parallax contributes to the accurate perception of self-motion (Hülemeier & Lappe, 2023). The simulation lasted for 2500 ms.

### Experimental Conditions

#### Crowd motion

The experiment comprised three crowd motion conditions (see Supplemental movies 1–16): static, natural locomotion, and only-articulation. In the static condition, the walkers resembled static figures and formed a rigid environment. They did not walk but kept their posture at a fixed position, thus having zero speed. In other words, the static condition only conveyed the participant's simulated self-motion. The natural locomotion condition presented the walkers naturally moving through the world and moving their limbs. This condition combined limb articulation with translation through the scene. The walkers in the only-articulation condition moved their limbs without physical translation through the scene, as if on a treadmill. Note that in each crowd motion condition, observer motion was simulated at a constant speed approaching the crowd.

#### Walker Type

Walkers could appear either normal or scrambled (see Supplemental movies 15 and 16). Scrambling spatially relocated the point positions within the minimum and maximum height and width coordinates of a normal walker, thereby dissolving the body form of the walkers and destroying any limb connections, but keeping the motion trajectories of each point the same (Cutting, 1981). The walkers were scrambled as a group, rather than indivudally, so that all scrambled walkers within a trial had the same scrambled shape. Scrambling was applied a new after each trial. By comparing data from normal and scrambled walkers, we were able to study the specific effects of limb articulation compared to the motion trajectories of individual points. Trials were blocked according to the walker type (normal vs. scrambled).

#### Articulation Rate

We tested three different articulation rates to see whether perceived crowd speed depended on articulation rate. To implement the different articulation rates, the original motion-tracking data was linearly interpolated to either 0.8 (slower) or 1.2 times (faster) than the originally recorded articulation rate of the motion-tracked walker (see Supplemental movies 2–7 for normal walker type, and movies 9–14 for scrambled walker type). In the natural locomotion condition, translation speed was adjusted by the same factor to maintain a natural connection between articulation and translation. Each walker in the crowd started from a different phase in the gait cycle. The static crowd did not articulate at different rates. In the experiment, we showed the static condition equally often as the other conditions.

### Procedure

We instructed participants orally and in writing about the experimental procedure, stimulus, and task. We told them that they would see a simulation in which they would approach a crowd of point-light walkers oriented collectively to the left or right. Their approach was at a constant speed and with different heading directions. The crowd's speed would change from trial to trial.

Participants’ task was to estimate the velocity of the crowd (not their self-motion velocity). The method of adjustment was used for the report of their estimate. Participants had to adapt the speed of a single walker in a way that the adjusted speed matched the perceived crowd speed. This single walker was presented in isolation at a distance of 6.5 m from the observer, without limb articulation, and without additional self-motion from the observer. The single walker did not articulate its limbs, regardless of whether the crowd articulated their limbs (only-articulation and natural locomotion) or not (static). This ensured that speed adjustments were not influenced by limb articulation. Moving the computer mouse along the horizontal line changed the speed from 0 to 2.8 times the natural translation speed. If the walker disappeared from the edge of the display, it reappeared on the other side. Once the participant had adjusted the speed to their satisfaction, they registered their response by pressing the left mouse button. After their response, the next trial started.

Participants completed a practice block without data collection and performance feedback. The practice block covered all stimulus combinations (18 trials) in randomized order. Data collection started after the practice block. An experimental block contained ten repetitions of all stimulus combinations in randomized order, i.e., 180 trials. Participants completed two experimental blocks of data collection. The entire experiment, including a short break between the experimental blocks, took about 45 min.

### Analysis Procedure

Our analysis examined the estimated crowd speed depending on the conditions. We used R (version 4.2.1) for the analysis. For data preparation, we calculated the average speed estimates per participant based on condition, walker type, and walker articulation rate. In natural locomotion, we determined whether participants correctly perceived different crowd speeds. Based on the speed estimates per participant, we, therefore, calculated a correlation between the estimated crowd speed and articulation rate (which directly corresponds to the true crowd speed). Moreover, we calculated the slope of the crowd speed estimates depending on the articulation rate using an ordinary least square regression model. We applied this correlation analysis analogously in the only-articulation condition to assess whether crowd speed estimates were influenced by the articulation rate or not. To further compare crowd speed estimates in the natural locomotion and only-articulation condition depending on the walker type, we employed a factorial design ANOVA. This ANOVA was based on the linear mixed model (LMM) framework for two reasons. First, LMMs are superior in handling non-normal data (Arnau et al., 2012). Our dependent variable was not normally distributed, thus, violating the assumption of ANOVA based on ordinary least squares regression models. Secondly, the mixed-modeling framework offers greater flexibility, accuracy, and power for repeated-measures data. The LMM achieves these benefits by accounting for fixed and random effects, as well as accommodating varying variances, covariances, and distributions (Jaeger, 2008; Kristensen & Hansen, 2004).

We fitted LMMs using restricted maximum-likelihood criterion and nloptwrap optimizer. Our model predicted the crowd speed estimates by the experimental conditions (natural locomotion vs. only-articulation) and their interaction with the walker types (normal vs. scrambled). Alongside these fixed effects, we incorporated participant id and the articulation rate as random effects.

By incorporating participant id as a random effect, we allowed for individual differences in their average crowd speed estimates (random intercept). Additionally, including the articulation rate as a random effect captures the variation in the effect of the average crowd speed (random slope). The model specification reads like this:

Lmer(crowdspeedestimate∼condition*walkertype+(1+articulationrate|id)

Standardized parameters were calculated by fitting the model on a standardized version of the dataset. We aligned the 95% confidence intervals (CIs) and *p*-values to the Wald approximation and labeled the effect sizes following Field's (2013) recommendations. In case of significant interaction effects, we post hoc analysis with *p*-value adjustments according to Holm (1979).

## Results

**Figure 2.**
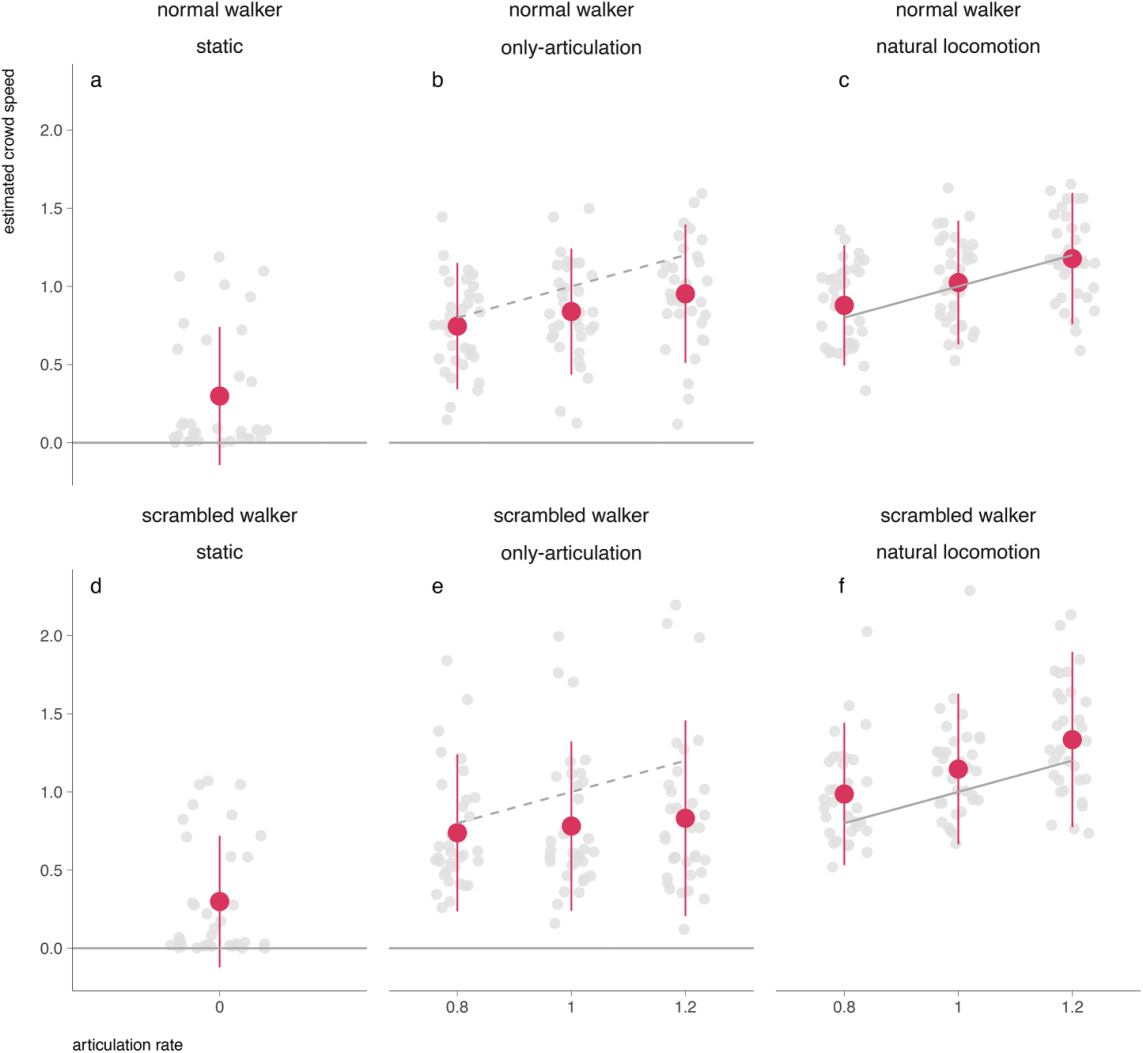
Crowd speed estimates per condition as a function of the articulation rate of the limbs. The top row (a–c) shows data for the normal walker type condition, while the bottom (d–f) row displays data for the scrambled walker condition. The data points for each participant are depicted in gray. The red dots represent the mean frequency across all subjects, with error bars indicating one standard deviation. The solid gray line shows the true physical translation speed of the crowd in each condition. The dotted gray line in (b) and (e) indicates the crowd speed which would be predicted from the articulation rate of the limbs.

Our research goal was to understand whether limb articulation may induce illusory lateral crowd speed percepts during self-motion. To describe the results and provide a coherent approach to our research question, we employed a two-step analytical procedure. We start with a descriptive analysis of the crowd speed estimates across conditions and walker types alongside Figure 2. This descriptive analysis provides a first impression whether participants could accurately perceive the absence (static) or presence (natural locomotion) of crowd speed, and how they perceived crowd speed in only-articulation, so when crowd speed was actually absent and the walkers merely articulated their limbs. We employed the scrambled conditions to differentiate between the limb articulation and the movement of individual points. We then proceeded with inferential analyses analyzing the interaction between the walker types and the only-articulation and natural locomotion conditions.

### Descriptive Analysis of Crowd Speed Estimates

We plotted participants’ average crowd speed estimates per condition (columns) and walker type (rows) (see Figure 2). As a reference, we added a solid line indicating the actual crowd speed. The dotted line in the only-articulation condition represents crowd speed estimates if crowd speed estimates were predicted by the articulation rate.

*Participants correctly perceived the absence of translation speed in the static condition:* In the static condition, participants adjusted the walker speed close to be close to zero, i.e., they accurately perceived that the walkers were stationary, and thus had no lateral crowd motion (Figure 2a and 2d). The crowd speed estimates for both scrambled and normal walkers in the static condition were similar in magnitude and close to zero (*M  *= 0.299 (both walker types), *SD *= 0.374 (scrambled) - 0.389 (normal)), as would be expected since both conditions present a rigid environment. The estimates neither significantly differed from each other nor did they have a large effect (*W *= 313.00, *p *= .800, *pcor* = .999; *r* (rank biserial) = .05, 95% CI [-.02, .02]).

*Crowd speed estimates were accurate for natural locomotion for normal walkers, but overestimated for scrambled walkers:* In the natural locomotion condition, the average crowd speed estimates for normal walkers appeared to be visually close to the true physical translation speed (Figure 2c). For scrambled walkers, crowd speed estimates appeared to be higher than their actual speed (Figure 2f). While the average error for normal walkers was *M  *= 0.027 (*SD *= 0.275, slope = 0.748), participants overestimated the speed of scrambled walkers by *M *= 0.156 (*SD *= 0.355) with an almost horizontal slope of 0.870.

The high accuracy for normal walkers gives evidence that the limb structure from the normal body form appears to provide important cues that enhance the accuracy of speed perception (Masselink & Lappe, 2015).

Based on the aggregated data per participant (see “analysis procedure”), we observed a positive and significant Spearman correlation between estimated crowd speed and articulation rate for both normal (*r *= .40, 95% CI [.22, .55], *p < *.001, one-sided testing) and scrambled walkers (*r *= .42, 95% CI [.24, .47], *p *< .001, one-sided testing).

*lllusory speed perception of the crowd in the only-articulation condition:* Figure 2b and 2e shows that the average crowd speed estimates in the only-articulation were larger than in the static condition (*M *= 0.299 for both walker types) for both normal (*M *= 0.846, SD = 0.321) and scrambled (*M *= 0.784, *SD *= 0.433) walker types. We additionally see that for normal walker types, the crowd speed estimates significantly depend on their articulation rate. Spearman correlational analyses revealed a significant positive relationship between estimated speeds and articulation rates for normal walkers (*r *= .27, 95% CI [.08, .45], *p *= .003, one-sided testing, slope = 0.518) but not for scrambled walkers (*r *= .06, 95% CI [-.14, .26], *p *= .259, one-sided testing, slope = 0.233). These findings suggest that the articulation rate influences the estimated crowd speed for normal walkers, but not for scrambled walkers. Yet even for scrambled walkers, participants perceived some crowd speed, albeit not depending on the articulation rate. The significantly larger variances observed for scrambled walkers in the only-articulation condition further highlight the uncertainty and difficulty participants faced in correctly and consistently interpreting the motion cues (*F*(101, 101) = 0.550, 95% CI [0.371, 0.815], *p *= .003).

### Comparing the Natural Locomotion to the Only-Articulation Condition Provides Evidence for Illusory Crowd Speed Perception

Comparing the natural locomotion and only-articulation conditions and their interaction with the walker types allows us to draw conclusions about the extent to which participants attributed their self-motion to the illusory lateral speed of the walkers (for details of the model specification see analysis procedure). Our model explains a substantial proportion of the variance (*R^2 ^*= .836) and the part solely related to the fixed effects is *R_mag._^2 ^*= .078. The intraclass-correlation coefficient is ICC = .822 and the residual standard deviation is σ = 0.214. Within our model, we indeed found a significant main effect for the crowd motion condition (*F*(1, 337) = 170.65, *p *< .001, $\eta^{2}$ (partial) = 0.47, 95% CI [0.32, 0.63]) with a large effect size. From the descriptive analysis, we know that speed estimates were in general higher in the natural locomotion than the only-articulation condition. Slower speed estimates in the only-articulation condition are reasonable from the subject's point of view. In the only-articulation scene, there is physically less motion than in the natural locomotion scene. The results suggest that the change in existing motion is incorrectly attributed to a slower crowd speed, rather than being correctly attributed to the complete absence of it. The main effect of the walker type did not reach significance (*F*(1, 337) = 2.46, *p *= .118, $\eta^{2}$ (partial) = -0.16, 95% CI [-0.32, −0.01]) and only showed a small effect size. In other words, the average crowd speed estimates did not differ significantly between walker types regardless the condition. Interestingly, the interaction effect between condition and walker type was significant (*F*(1, 337) = 10.27, *p *< .001, $\eta^{2}$ (partial) = 0.50, 95% CI [0.28, 0.71]) with a large effect size. Post hoc analyses comparing the crowd speed estimated between crowd motion conditions and walker types found overall significant differences (*p *< .001), except for the difference between normal and scrambled walker types in only-articulation (*M_diff _*= 0.06, *p *= .164). This latter comparison reflects the descriptive findings showing only slight differences between walker types. Also in line with the descriptively observed overestimation for scrambled walkers, the difference between normal and scrambled walker types within natural locomotion was significant (*M_diff _*= -0.13, *p *< .001). Generally speaking, the post hoc comparisons showed larger crowd speed estimates in natural locomotion than in only-articulation, regardless of the walker type (*M_diff _*= 0.18–0.37, *p *< .001).

Summarizing the comparisons between crowd motion conditions and walker types, we see that the biological motion of the normal walker leads to more accurate speed estimates in natural locomotion compared to overestimations for scrambled walkers. In the only-articulation condition, limb articulation evokes the perception of the theoretically corresponding crowd speed. As scrambled walkers in the only-articulation condition also create an impression of crowd speed, we conclude that the information in the motion trajectory of the individual points also contains cues to attribute a speed to the crowd, but more so for the normal walker because of the significant relation between the crowd speed estimate and the articulation rate. These results together suggest that a mechanism similar to the backscroll illusion (Fujimoto, 2003; Fujimoto & Sato, 2006; Fujimoto & Yagi, 2008) causes a crowd speed perception of the walkers that physically is non-existent.

## Discussion

We explored whether limb articulation of a crowd causes an illusory perception of the crowd's speed when one is approaching the crowd. We also investigated the extent to which this illusory crowd speed perception is linked to the limb articulation of the walking human body. We simulated self-motion at a constant speed toward a crowd of point-light walkers collectively facing to either the left or right side. Their form appeared as either normal or scrambled (see Figure 1), i.e., with or without proper limb configuration. Further, the crowd could be static (neither speed nor limb articulation), or move through the scene with the natural articulation of the arms and legs, or—in the only-articulation condition as our condition of interest—display the walkers only articulating the limbs without actually moving through the scene. Only the natural locomotion condition contained physical motion to the side (from the observers’ perspective). Participants reported their perceived crowd speed.

Our study was motivated by previous experiments on heading perception in the presence of biological motion. Biological motion violates the rigidity assumption that underlies heading perception and causes heading biases (Hülemeier & Lappe, 2020; Koerfer & Lappe, 2020; Riddell & Lappe, 2017, 2018; Riddell et al., 2019). While the combination of limb articulation and body translation in natural locomotion allows surprisingly accurate heading perception despite massive perturbations in the flow field (Hülemeier & Lappe, 2020; Riddell & Lappe, 2018), significant heading errors occur when only limb articulation without translation is presented (Hülemeier & Lappe, 2020; Riddell & Lappe, 2018). These heading biases might be explained by erroneous self-motion perception that results from attributing an illusory motion to the crowd based on its articulation (Hülemeier & Lappe, 2020, 2023). The present study aimed to test whether such an illusory crowd motion is perceived.

The static condition provided a baseline that is absent of walker translation and limb articulation. Motion signals arose exclusively from the simulated observer motion. Therefore, the static condition allows us to assess whether participants are in general able to detect the absence of crowd motion. The walker type should not matter for this question as both walker types produce a static environment well suited for self-motion estimation. Indeed, we found that participants accurately assessed the absence of crowd speed regardless of the walker type.

The natural locomotion scene provided a baseline for the case in which the crowd moved at a speed that matched their limb articulation. In this condition, the motion signals of the dots in the scene combine the simulated observer motion toward the crowd with the crowd moving to the side. The condition, thus, measures whether participants can estimate the crowd speed from the natural combination of limb articulation and translation despite the simulated forward self-motion. From previous studies (Masselink & Lappe, 2015), we know that humans are able to deduce the matching translation speed from limb articulation. We also know that heading estimates are quite accurate in this condition (Hülemeier & Lappe, 2020; Riddell & Lappe, 2018). Hence, we expect accurate crowd speed estimates. Indeed, we found that participants accurately estimated crowd speed in the natural locomotion condition with normal walkers.

The only-articulation condition was our main experimental condition. Here, two potential patterns of result were plausible. Let's first look at the situation from a pure optic flow perspective. Since the crowd does not move across the scene, the violation of the rigidity assumption is more minor than in the natural condition. In fact, the violation results only from the back-and-forth motion of the limbs, which, in essence, creates balanced noise. Hence, we would expect high variance but no bias, i.e., on average a correct estimation of zero crowd speed. Thus, if participants were able to accurately discern the absence of crowd motion through the scene, their crowd speed estimates should align with those in the static condition. Alternatively, participants might resort to limb articulation to estimate crowd speed based on their knowledge of the natural connection between limb articulation and walker translation (Masselink & Lappe, 2015). In this case, their crowd speed estimates should resemble those of the natural locomotion condition. Crowd speed estimates would then point to an illusory crowd motion perception since the crowd in this condition did not move through the scene.

The results with normal walkers showed that crowd speed estimates in the only-articulation condition resembled those of the natural locomotion condition rather than static condition, thus favoring the second alternative. Precisely speaking, participants reported crowd speed estimates that were lower than in the natural condition but well correlated with the articulation rate. We, thus, concluded that the limb articulation produces an illusory percept of crowd speed. The overall lower reported speed estimates may either indicate that the illusory percept is not 100%, or it may be because the only-articulation condition contains physically less motion than the natural locomotion condition, as the walkers do not actually move across the scene.

In a second set of conditions, we compared normal walkers and scrambled walkers to assess the role of limb articulation vs. the motion of the individual points. Biological motion perception is derived from an analysis of the changing shape of the human body during articulation (Beintema & Lappe, 2002; Giese & Poggio, 2003; Lange & Lappe, 2006). Additional information is also available in the motion and trajectories of individual points (Giese & Poggio, 2003; Troje & Westhoff, 2006). Scrambled walkers destroy the body form and hence the limb structure but retain the motion trajectories of the individual points (Cutting, 1981). Previous work on heading estimation when moving toward a crowd had used scrambled walkers to investigate whether heading biases are specific to the limb articulation of biological motion (Hülemeier & Lappe, 2020). In that study, the heading bias was larger for scrambled than for normal walkers in the natural locomotion condition. This indicates that biological motion contributes to accurate heading perception when articulation and translation of the crowd match. For the only-articulation condition, on the other hand, large heading biases occurred for normal walkers, whereas scrambled walkers produced only slight biases. This suggests that limb articulation along with the biological form produces the bias. For the present study, those results would predict that normal walkers should produce more accurate crowd speed estimates in the natural condition compared to scrambled walkers, while scrambled walkers should produce less illusory crowd speed in the only-articulation condition.

Both predictions were borne out in the data. In the natural locomotion condition, the crowd of normal walkers led to accurate estimates of crowd speed, whereas the crowd of scrambled walkers produced estimates that were too high. In the only-articulation condition, the crowd of normal walkers led to an illusory crowd speed that depended on articulation rate, whereas the crowd of scrambled walkers resulted in crowd speed reports that did not depend on articulation rate and were overall smaller.

One might argue that the concept of articulation rate cannot be applied to the scrambled walkers since the connections between the joints are destroyed and limb articulation is defined as the motion of one joint relative to another. This is certainly correct, but the correlation between articulation rate and estimated crowd speed in the natural locomotion condition with scrambled walkers shows that some information on articulation rate is also available for the scrambled walkers. This likely derives from the rhythmicity of the trajectories of the individual points, most notably in the trajectories of the feet (Troje & Westhoff, 2006). These trajectories convey the step rhythm, and, by extension, a measure of the articulation rate of a walker.

The motion trajectories of the feet might also provide an explanation for the question of why there is an illusory percept of crowd speed at all in the only-articulation condition. It is known that the lower legs provide the most important information about walking motion in point-light walkers (Mather et al., 1992; Theusner et al., 2014; Troje & Westhoff, 2006). For example, even for scrambled walkers, the trajectories of the feet allow observers to indicate the direction in which the walker is facing (Troje & Westhoff, 2006). Moreover, the trajectories of the feet provide important information also for heading toward a crowd, since the phases in which they touch the ground provide stable information about optic flow, self-motion, and, by extension, the motion of the crowd (Riddell & Lappe, 2018). However, from our data, it seems that this information is not sufficient to provide a dependency on articulation rate in the only-articulation condition.

Our study was motivated by previous research on heading perception in the presence of biological motion, a task we regularly encounter in daily life. While biological motion clearly disrupts the rigidity assumption that is usually made in the analysis of optic flow, recent research has shown that heading perception is nonetheless quite accurate in the presence of biological motion (Hülemeier & Lappe, 2020, 2023; Riddell & Lappe, 2018). One reason for this lies in the above-mentioned stable phases of the feet when touching the ground, which provide brief glimpses of a stable scene (Riddell & Lappe, 2018). Another reason lies in the information conveyed by biological motion about the translation of the crowd. While this information is helpful when limb articulation and crowd speed match, its contribution is seen most dramatically in the heading biases that are reported when articulation and crowd speed do not match (Hülemeier & Lappe, 2020, 2023). These biases can reach several tens of degrees of visual angle. The present study shows that they result from an illusory percept of crowd speed that is produced by limb articulation. Even though the crowd remained static in the scene, participants reported that it appeared to move within the scene at a speed that matched its articulation rate. This illusory crowd speed is consistent with an illusory motion of the background when biological walking-in-place is presented in front of a background (Fujimoto, 2003; Fujimoto & Sato, 2006; Fujimoto & Yagi, 2008). Complementing this backscroll illusion, we further found that even the natural motion of individual points, such as the feet, can induce an illusory motion percept as substantiated by crowd speed estimates for scrambled walkers. Taken together, our study provides further evidence for the different ways in which information from biological motion contributes to the perception of self-motion in interaction with other people.

## Supplemental Material

**Figure:** Video 1.

**Figure:** Video 2.

**Figure:** Video 3.

**Figure:** Video 4.

**Figure:** Video 5.

**Figure:** Video 6.

**Figure:** Video 7.

**Figure:** Video 8.

**Figure:** Video 9.

**Figure:** Video 10.

**Figure:** Video 11.

**Figure:** Video 12.

**Figure:** Video 13.

**Figure:** Video 14.

**Figure:** Video 15.

**Figure:** Video 16.
